# Phage Prevalence in the Human Urinary Tract—Current Knowledge and Therapeutic Implications

**DOI:** 10.3390/microorganisms8111802

**Published:** 2020-11-17

**Authors:** Maciej Żaczek, Beata Weber-Dąbrowska, Ryszard Międzybrodzki, Andrzej Górski

**Affiliations:** 1 Bacteriophage Laboratory, Ludwik Hirszfeld Institute of Immunology and Experimental Therapy, Polish Academy of Sciences, 53-114 Wrocław, Poland; maciej.zaczek@hirszfeld.pl (M.Ż.); beata.weber-dabrowska@hirszfeld.pl (B.W.-D.); ryszard.miedzybrodzki@hirszfeld.pl (R.M.); 2 Phage Therapy Unit, Ludwik Hirszfeld Institute of Immunology and Experimental Therapy, Polish Academy of Sciences, 53-114 Wrocław, Poland; 3 Department of Clinical Immunology, Transplantation Institute, Medical University of Warsaw, 02-006 Warsaw, Poland; 4 Infant Jesus Hospital, Medical University of Warsaw, 02-005 Warsaw, Poland

**Keywords:** phage therapy, urinary tract, uropathogenic bacteria, urinary tract infections (UTIs), antibiotic resistance, microbiome, urobiome, phage circulation

## Abstract

Recent metagenomic analyses imply an immense abundance of phages in the human body. Samples collected from different sites (lungs, skin, oral cavity, intestines, ascitic fluid, and urine) reveal a generally greater number of phage particles than that of eukaryotic viruses. The presence of phages in those tissues and fluids reflects the paths they must overcome in the human body, but may also relate to the health statuses of individuals. Besides shaping bacterial metabolism and community structure, the role of phages circulating in body fluids has not been fully understood yet. The lack of relevant reports is especially visible with regard to the human urobiome. Certainly, phage presence and the role they have to fulfill in the human urinary tract raises questions on potential therapeutic connotations. Urinary tract infections (UTIs) are among the most common bacterial infections in humans and their treatment poses a difficult therapeutic dilemma. Despite effective antibiotic therapy, these infections tend to recur. In this review, we summarized the recent data on phage presence in the human urinary tract and its possible implications for health and disease.

## 1. Introduction

Our bodies are inhabited by trillions of indigenous microorganisms. In addition to this statement, the idea that all bacteria, fungi, viruses, and other microorganisms impact our health has been growing as well. For instance, it has been suggested that alterations in the gut microbiome can have a direct effect on colon cancer development [1]. Phages constitute one of the major parts of the human microbiome. They have been found in almost every niche of the human body. Phages have evolved various strategies to overcome the epithelial barrier [2]. Microscopic methods helped to reveal that total counts of bacterial viruses in human feces, cecal content, and colonic mucosa are as high as 10^9^–10^10^ virus-like particles per gram [3]. Other sources estimate phage count as high as approximately 10^15^ particles in the human gut [4]. Thus, we can easily assume that phages play an important role in shaping composition of human microbiome and, indirectly, affect our overall health status. Phage importance is evidenced, among others, by the vast range of mutual bacteria–phage interactions. One could predict that their widespread predation triggers adequate bacterial response in the form of molecular mechanisms, such as restriction modification system (RM system), providing defense against exogenous DNA [5,6], pseudolysogenic states, a major factor selecting phage-resistant mutants [7], or the newly described bacteriophage exclusion (BREX) system that blocks phage DNA replication [8]. Unlike the RM system, BREX takes place during the late phases of infection, and exploits gene coding phage proteins, including those already injected into the bacterial host. In a similar way, phage presence may initiate immunological response of higher organisms through the induction of antiphage antibodies [9,10,11], and even impact the expression of immunologically important genes, which was recently shown in an example involving Caco-2 cells [12]. The fact that over 80% of healthy individuals were found to have *Escherichia coli* antiphage (anti-T4) antibodies in their sera reflects vast and continuous interplay between phages and cells of the human immune system [13].

Human body fluids, such as a serum, urine, and, to a lesser extent, saliva, constitute one of the largest sources of biomarkers. The analysis of these body fluids may be supportive in the diagnosis and prognosis of a disease. A particular component in body fluids can, thus, be considered as a marker helpful in detecting a particular disease. In such a way, the presence of phages in those fluids may sometimes be associated with the health status of the patient. For instance, gut barrier disruptions resulting from inflammation or other diseases may favor phage penetration to the blood stream. Moreover, phages can infect gut microbiota and lead to the disease called leaky gut. This phenomenon was explained by Tetz et al. in an experimental rodent model [14]. The same group discovered *Shigella* and *Staphylococcus* phages in human cerebrospinal fluid of patients with multiple sclerosis. Such a phenomenon suggests phage transports to the cerebrospinal fluid and the brain, which would not be possible in healthy subjects as these regions are considered “sterile” [15]. Bachrach et al. [16] observed a relatively high prevalence of *Enterococcus faecalis* phages in 22% of the saliva of random donors. Such prevalence of *E. faecalis* phages in oral cavity suggests possible continuous interplay between phages and bacteria whose range is limited mostly to the roots of the teeth, deep below the gum line.

While phages have been found to have a significant influence in various microbial environments, little is known about their presence and impact on the urinary tract in humans. Nonetheless, phage prevalence may also be associated with unfavorable effects. Vast prevalence of phages in different sites of the body could be responsible for horizontal gene transfer between phage and bacterial genomes enhancing survival of bacteria through acquiring new beneficial functions, such as resistance to antibiotics. It has already been pointed out by Thannesberger et al. [17] on the example of human oropharynx and the urinary tract. In line with the above, the aim of this article is to find an answer to what is the role of urinary microbiome with phages as its significant component.

## 2. Human Urobiome

Although urine samples of healthy subjects had been considered sterile, new diagnostic techniques that do not involve culturing of microorganisms shed new light on this belief [18]. Analyzing the microbiome of human urinary tract requires a careful approach as the urine specimen collection technique will influence the outcome [19]. The authors suggest that, in women, voided urine specimens should be considered genitourinary specimens as there is only one direction for emptying the bladder, and it involves passage through vulvovaginal part of the genitourinary system and its microbiome. Bajic’s work also highlights the difference between voided and catheterized specimens. The lower urinary tract microbiota was found in 98% of voided specimens whereas only in 39% of catheterized ones [20].

Among bacterial species inhabiting a genitourinary niche, one could mention *Lactobacillus, Gardnerella, Prevotella, Sneathia, Atopobium, Enterococcus, Streptococcus,* and *Enterobacteriaceae* in women, and *Lactobacillus, Sneathia, Veillonella, Corynebacterium, Prevotella, Streptococcus* and *Ureaplasma* in men [21]. Bladder microbiome has a low biomass relative to the vaginal microbiome and is dominated by *Lactobacillus, Gardnerella,* and *Streptococcus* [19]. Moreover, it has been demonstrated that uropathogenic *E. coli* (UPEC) can invade and replicate within the bladder cells to form intracellular complexes, making them resistant to antibacterials and the host immune system [22]. The composition of the urobiome, similar to that of the intestinal model, changes in an age- and health-dependent manner. While *Lactobacillus crispatus*, along with *Veillonella, Streptococcus,* and *Corynebacterium* were found only in healthy individuals, the *Streptococcus* genus was found more frequently in patients with bladder cancer [19,21]. Such health-dependent alterations in urinary microbiota are also described in diabetes patients by Liu et al. [23]. The authors observed significantly lower association between the presence of *Lactobacillus* in the group of patients with diabetes and hypertension, and higher in the diabetes only, and diabetes with hyperlipidemia cohorts. Recent studies imply that composition of urinary microbiome may be responsible, at least partially, with age, body mass index, and concomitant therapies, for urinary incontinence in women [18]. The prevalence of *Lactobacillus gasseri* in the bladder was shown in women with urge incontinence, while *L. crispatus* was typical for those without symptoms. [24]. It was shown that human microbiome is inherently dynamic and varies across populations with various health conditions. It is no different with urobiome. Price et al. [25] found that urobiome composition changes during menstruation and vaginal intercourse, but is relatively stable during health. Similar observations have been noted by Ghose et al. [26] who found that viromes of urine, saliva, and feces were of relatively high diversity, in contrast to breast milk, body fluids, plasma, and cerebrospinal fluid (CSF). The existence of a link between bladder microbiota and the degree of urinary tract infections (UTIs) in males was showed by Bajic et al. [20]. The presence of lower urinary tract microbiota was confirmed in nearly 60% of patients with severe UTI symptoms.

It is worth mentioning that bacteria that are part of human urobiome can exhibit unusual physiological properties, leading them to survive under unfavorable conditions. Mickiewicz et al. [27] isolated cell-wall deficient *E. coli* strains (L-forms) from urine samples collected from elderly patients with recurring UTIs following antibiotic treatment. Moreover, bacteria were able to rebuild their cell wall after completion of antibiotic administration. During the L-form phase, bacteria were not only able to survive, but also retained their ability to divide. The authors suggest that this phenomenon may play a crucial role in recurrence of UTIs in patients.

## 3. Phages as Part of Urinary Microbiota

Data describing virus presence in the urinary tract are much scarcer, especially in the context of prokaryotic viruses. Thus, the role of phages in the urobiome, so-called U-phages, remains largely unknown. This may be surprising given the fact that a vast abundance of phages occurs also in the urinary tract. Among 30 tested urinary samples collected from healthy women, as well as women with overactive bladders, viral genomes were detected in 27 of them [28]. Brown-Jaque et al. [29] detected infectious tailed phages in >45% of ascitic fluid and urine samples. Notably, part of the tested samples was contaminated during the collecting process, as indicated by the authors themselves. This phenomenon addresses the aforementioned issues regarding the urine specimen collection process. A particular emphasis should be put on appropriate collection technique and the risk of possible contaminations.

As early as 1925, Larkum noted the presence of phages in about 25% of the urine samples from patients having urinary tract infections [30]. Although Larkum found bacteriophages exclusively in the urines from individuals having acute infections, one must be aware that diagnostic assays available a hundred years ago were facing a lack of sensitivity that is a standard these days. Malki et al. [31] reported about seven *E. coli* phages isolated from the bladders of four adult women with urge urinary incontinence. However, their potential impact on the disease remains unclear.

Analysis of homologous viruses shared between specimen types conducted by Ghose et al. [26] revealed that all three phage families, myoviruses, siphoviruses, and podoviruses, were present in feces, urine, and saliva, but in addition, each of these virus types was also present in such samples, as breast milk, CSF, or body fluids. Interestingly, only 4 of the 20 urine viromes were identified implying the presence of a large count of still unknown sequences. The parallel conclusion was obtained by Miller-Ensminger et al. [24]. In their study, the majority (57%) of phages isolated from the bladder had unique sequences that could not be compared to any known phage genomes. The occurrence of completely new genomes was also noted by Santiago-Rodriguez et al. [32]. Out of 20 tested urine samples collected from healthy individuals and the patients suffering from UTIs, only approximately 30% of the samples could by identified by their contigs. Interestingly, the 27% of identified contigs indicated the presence of viruses with the majority (>99%) representing phages. Because of the high proportions of integrases, the authors predicted that temperate phages constituted a significant part of the phage population. The authors estimated the total phage count in investigated samples at 10^7^ pfu/mL of urine sample, which was an order of magnitude lower than found by the same authors in human saliva. Letarov et al. [33] provided data on relatively low phage concentration in urine (10^1^–10^2^ pfu/mL) which could be a result of poor phage stability in urine. The authors suggest that phage particles colonize the urine not only via filtration through the Malpighian tufts, but also through translocation from the blood into the canal of epithelium, i.e., a special type of eukaryotic cells covering the body or lining a body cavity that constitute a barrier against the external environment [15].

In terms of shifts in viral communities within urobiome, there are contradictory reports focusing on this phenomenon. The aforementioned Santiago-Rodriguez et al. [32] noted a well-balanced viral community in the urinary tract, regardless the health status of individuals. As one would expect, such differences were noted in bacterial communities in samples collected from the UTI-diseased individuals [23]. Contrary to these findings, Miller-Ensminger et al. [24] were able to isolate *Actinomycetaceae* temperate phages from strains colonizing women with overactive bladders, but not from healthy subjects.

## 4. The Role of Prophages within Urobiome

Although the urinary tract is inhabited by both lytic and temperate phages, researchers are beginning to explore the signals that contribute to prophage induction in the microbiota. Answering the question of what factors are responsible for switching between phage life cycles could contribute to a better understanding of phage prevalence in different sites of the body. So far, we know that certain antibiotics, diet, and health status may contribute to prophage induction [34]. Recent reports suggest that phages can gain information on available hosts in their vicinity and, based on that information, are able to switch to the lytic cycle [35]. It is worth it to mention quorum sensing, a phenomenon that leads to gene expression among bacteria in response to their density within a particular niche. This mechanism was recently described in *Bacillus* phages [36]. Investigated phages were able to release during infection a peptide, the concentration of which were later used by other phages to determine switching between cycles. Miller-Ensminger et al. suggest that the prophage population in the urinary microbiota is large, similar to the composition of other microbiotas in the human body. This assumption is in line with the abovementioned report by Santiago-Rodriguez et al. [32] who identified phage-like sequences corresponding to temperate phages in more than 99% of the urine samples. In addition, among 181 bacterial isolates from the bladder of healthy and UTI’s diseased women, the majority (86%) harbored one or more lysogenic phages [24]. It has been already described that carrying a prophage entails a plethora of benefits, such as protection of bacterial cells from phage superinfection, an increase of bacterial fitness through phage genes or even management of bacterial virulence [17,24].

Recently, a new filamentous phage UPϕ901 was isolated from an *E. coli* strain found in clinical urine sample [37]. This phage, a member of *Inoviridae* family, is not a typical temperate phage and can maintain long-term replication cycles without lysing its host. According to Shapiro and Putonti [37], filamentous phages can have significant effects on their hosts’ virulence through altering bacterial motility, biofilm formation, and carrying unfavorable genes, among others. However, the role of the newly described group of phages (named by the authors UPϕ viruses), despite their vast abundance among *Enterobacteriaceae,* remains unclear. It is possible that these phages alter bacterial urobiome composition without changing bacterial virulence. As mentioned above, filamentous phages have been shown to encode genes responsible for bacterial virulence. Waldor and Mekalanos [38] described cholera toxin and toxin-coregulated pili of *Vibrio cholerae* that are essential in *V. cholerae* intestinal colonization. Both factors are related to a filamentous phage (CTXphi). This discovery led to the exploration of new filamentous phages associated with *V. cholerae* that might be involved in horizontal gene transfer [39]. Another 17 distinct groups of *Streptococcus anginosus* prophages were identified from catheterized urine samples in women [40]. *S. anginosus* is currently considered an emerging pathogen. Similar to previously described reports regarding extensive prevalence of prophages within urinary microbiota, the authors revealed the presence of prophages in all bacterial isolates, some of which were able to initiate the induction in bacteria.

Miller-Ensminger et al. [24] note that some phages integrated into the chromosome of bladder bacteria may be unable to enter the lytic cycle. A prophage isolated from its host *E. coli* UMB0901 was not able to lyse several *E. coli* bladder isolates but was lytic against laboratory strains of *E. coli* C and K-12. On the other hand, Garretto et al. [41] following analysis of publicly available *Gardnerella* genomes found that phage infections were common among bacteria inhibiting urinary tract. Miller-Ensminger et al. [24] conclude that temperate phages may be involved in controlling the urinary microbiota stability. The same authors isolated from the urinary tract numerous *Lactobacillus jensenii* prophages, suggesting that their role in urinary microbiota could be protective. Previous reports described the protective role of *Lactobacillus johnsonii* phage endolysin against *Clostridium* in the gut [42]. Recently, Crawford et al. [43] revealed that the genome of antibiotic resistant *E. coli* UMB1353 isolated from the female urinary tract contains one intact *Enterobacteriaceae* P2-like phage.

## 5. Phages in the Development and Treatment of UTIs

Urinary tract infections, one of the most common bacterial infections, challenge physicians around the globe as they are difficult to heal and tend to occur. This type of disease concerns approximately 150 million people annually worldwide [44]. Women are more prone to UTIs, mostly due to their anatomy, and they can suffer from the disease in all age groups, starting from school-aged girls to elderly population [45]. The only exception is among uncircumcised boys during the first few months of their lives, when the prevalence of UTIs is higher in boys [46]. The most popular pathogen causing urinary infections is UPEC responsible for both complicated (50–65%) and uncomplicated (75–85%) UTIs [44]. Recent reports show that the major source of UPEC strains is the gastrointestinal tract. The authors observed that alterations in fecal bacterial communities of adults may be responsible for the development of UTIs in subsequent stages of the disease. By analogy, patients treated with fecal transplant exhibited less frequent recurrence of UTI [46]. There are possibly three major routes of infection within urinary tract proposed in the literature: intestinal bloom of bacteria followed by bladder colonization; reinfection from an external source (e.g., urinary catheter); bacterial persistence within the genitourinary tract [46]. Some sources indicate that single insertion of a catheter significantly decreases rate of bacterial colonization, while catheters with open-drainage systems result in bacterial contamination in almost 100% of the cases [47,48].

Based on high diversity of urinary microbiome, Mueller et al. [49] hypothesize that UTIs are caused by spectrum of urinary dysbiosis, rather than invasion by a single pathogenic strain. Interestingly, expansion of phage populations during dysbiosis has been showed in a murine model of colitis [50]. Convergent opinion comes from Kline and Lewis [51], who described bacterial synergy in experimental models of polymicrobial UTIs. Such synergy may be induced by strains that either reside in or are exposed to the urinary tract. One could imagine a prominent role of phages in proposed mechanisms as their presence is inevitably associated with bacterial populations. Their role could be favorable or negative with likely therapeutic implications. The mutual balance and interplay between phages an bacteria and its role in pathogenicity was demonstrated by Liao et al. [52]. The authors found that pretreating urinary catheters with benign *E. coli* HU2117 strain and lytic *Pseudomonas* phage ΦE2005-A would prevent *Pseudomonas aeruginosa* biofilm formation on catheters—a crucial aspect in the pathogenesis of catheter-associated UTIs. Notably, phages applied alone were not able to kill *P. aeruginosa* cells. However, phage–host interactions perhaps are more complicated than most laboratory studies and one must be aware that different phages can affect their bacterial hosts differently [53]. Such interactions, considered together with human immune response, overall patient health status, route of administration, and a plethora of other molecular and clinical factors, gives us an idea of unprecedented complexity of the phage therapy model, and it is difficult to predict outcome. In fact, only carefully planned clinical studies will find an answer regarding the truly therapeutic effect of phage treatment. Although first clinical trials have already been described in the literature, we will have to wait for final and unambiguous results [54].

Intriguing data come from murine model of UTIs induced by *Cronobacter* [55]. Besides phage lytic activity, resulting in 70% reduction of bacterial count, the authors noted that phages were able to decrease oxidative stress in UTIs. Such observation is of high importance in pathogenesis where oxidative damage of tissues plays an important role.

The aqueous environment of the urinary tract makes it a convenient niche for therapeutic applications. Water favors multidirectional motion of phage particles and increases the possibility of contact between them and their bacterial hosts [56], whereas a low-moisture environment significantly reduces phage mobility and, indirectly, their lytic action. Phages deprived of mobility are unable to attach to specific receptors on bacterial surface, which results in an inability to disrupt the bacterial cell wall and initiate the infection [57]. From an anatomical point of view, the urinary bladder is easily accessible via catheterization (in some cases patients are even taught self-catheterization technique) [58] and any bacterial residues can be, at least in some cases, efficiently removed by urination or irrigation. Further, phages were proven to be effective in biofilm reduction and seem to be persistent to washout during urine voiding. Although phage therapy in UTIs faces obstacles that are common in phage treatment in general such as resistance to phages or narrow phage host range [59], their detailed analysis is beyond the scope of this review. The recent attempts of using phages in the treatment of UTIs, both in vitro and in vivo, along with treatment outcomes are summarized in Table 1.

### 5.1. Urinary Phages (U-Phages) and Local Immune Response to Infection

Data regarding the immune functions of the lower urinary tract are still scarce. Although several lines of defense exist in the urinary bladder (a mucus layer, antimicrobial peptides, immunoglobulins, resident, and recruited immune cells), UTIs are frequently recurrent, suggesting that those defense mechanisms are inadequate. Therapeutic strategies to boost the immune system might be efficient in preventing UTI.

Moreover, attenuating inflammation would also be useful. However, non-steroidal anti-inflammatory agents may promote pyelonephritis [67]. Therefore, the potential role of U-phages might also be considered in this context. In fact, phages could contribute to local defenses within the urinary tract in a number of ways: (a) phages may induce beta-defensins known to have a role on the protection of UTI [67,68]; (b) most phages assayed do not downregulate Toll-like receptor 4 (TLR4) expression [68], which is relevant in view of the findings suggesting that normal TLR may protect against UTI [67]; (c) neutrophil-derived reactive oxygen species (ROS) may damage the urothelium promoting exfoliation [67], while phages are known to have anti-inflammatory properties downregulating ROS production by neutrophils [69]. The latter activity of phages has been confirmed by Tothova et al. [55], who have shown that phage therapy of mice with UTI attenuates the expression of pro-inflammatory cytokines.

### 5.2. Phage Translocation and Stability within the Urinary Tract

The ability of phages to maintain microbiome stability and to control bacterial hosts depends on their lytic properties. Interesting data on phage stability in the urinary tract are provided by Goetsch et al. [70]. The dsDNA *E. coli* bacteriophage T3 spiked into various hydrolyzed urine samples turned out to be much more stable than human polyomavirus (BKPyV), despite having the same genome type. Possible disulfide bonds in the BKPyV capsid make it more susceptible to inactivation. Chandran et al. [71] tested the survival rate of MS2 coliphage in human urine samples. Both fresh and diluted urine significantly deteriorated phage viability. Overall, fluctuations in pH levels had devastating effect on coliphage MS2 viability. These data indicate the possible continuous flow of phages, at least to some extent, into the urinary bladder that can later be isolated from urine samples.

Tan et al. [53] did not observe deleterious effect of urine on suspended phage particles when testing two *Klebsiella* phages against nine carbapenemase-producing *K. pneumoniae* isolates from two elderly patients with UTIs. Pereira et al. [60] suggested that phages against urinary tract bacteria are well adapted to persist in the urinary tract niche, which is characterized by rather low pH values (according to the American Association for Clinical Chemistry, the average value for urine pH is 6.0, but it can be even as low as 4.5). In the authors’ experiment, *E. cloacae* phages remained stable with preserved lytic activity for at least 12 h in urine samples.

In an investigation by Cardoso et al. [72], biological evaluation of radiolabeled *P. aeruginosa* phage PP7 after intravenous injection has been performed in mice. Within 3 h, phages were found mostly in urine bladder, irrespective of the overall condition (normal mice vs. infected mice). In all groups, phage accumulation in urine bladder reached 50% of the injected dose. Holman et al. [73] noted that a total urinary excretion in mice at 5 and 30 min was 17.2 ± 5.3% and 31.3 ± 2.7% of injected phage dose, respectively. Although radiolabeling could impact phage infectivity and activate nonspecific hepatic and splenic uptakes, the authors revealed phage concentrations as high as >10^8^ pfu/mL in urine samples [73]. Undoubtedly, the issue of phage journey in the urinary tract habitat and the factors that regulate this process require further investigation.

Studies conducted in humans provide additional data in terms of phage penetration and their therapeutic efficacy in urinary tract [74]. The most effective routes of administration of phage preparations in 22 women with genital or urinary tract infections were the intravaginal route and the oral route, both of which resulted in 50% rates of good responses to treatment. A relatively high rate of patients with favorable outcome following oral application suggests that phages effectively penetrated to the site of infection from gastrointestinal tract. In 29 men with genital and/or UTIs, 60% of patients achieved good response to phage therapy after intrarectal administration, and 33% in cases where phages were applied in combination (intrarectal and topical application). Intrarectal application of phage-based therapeutic turned out to be highly effective in recent case study from Poland [75] describing treatment of UTI in a 60-year-old kidney transplant recipient. A remission of clinical symptoms was achieved which persisted over 4 years after removal of the left kidney and 5.5 years after kidney transplantation. However, it must be highlighted that the patient underwent combined therapy with meropenem to which pathogenic ESBL-producing *K. pneumoniae* was fully susceptible. Obviously, findings presented above are relevant only for therapeutic phages, while naturally occurring ones may exhibit a variety of adaptation mechanisms, as stated above, to persist within the urobiome. Such mechanisms include the ability to avoid immune mechanisms of the host which has been demonstrated by Hodyra-Stefaniak et al. [76].

The review of recent literature drew our attention to an increasing number of reports describing phage treatment of biofilm formation in urinary catheters [77,78], which constitute a unique environment for microorganisms. Because catheter-associated urinary tract infections (CAUTIs) are common hospital-associated infections (HAIs), and the efficacy of antimicrobial urinary catheters is poorly studied, such attempts are fully understandable. Current reports indicate that phage therapy could be effective in catheters with preserved phage propagation despite continuous washout. It has been shown that phages are able to embed in the mucosa, which can protect them from washout during urine voiding [79]. In accordance with the above, a considerable reduction in biofilm formation was found after “coating” indwelling catheters with bacteriophages specific for *P. aeruginosa*, *E. coli*, and *P. mirabilis* [80]. Intriguingly, Chibeu et al. [61] showed that phages might reduce biofilm formed by uropathogenic *E. coli* in a non-dose-dependent manner, meaning that low titers of the phages were as effective as using a higher titer in eradicating established biofilms.

## 6. Discussion

The discovery of a virome in the body fluids such as CSF is a clear indication that none of the niches in the human body may be considered sterile. To date, viromes have been identified in saliva, dental plaque, feces, skin surfaces, breast milk, urine, lungs, blood, the vagina, and abovementioned CSF [26]. Moreover, human body consists of so-called dark matter, which has been poorly investigated so far. Among the plethora of phages identified in 111 samples collected from human body fluids, Pacifico et al. isolated phages belonging to newly described subfamilies, such as *Tunavirinae*. None of these phages lysed bacteria isolated from the same samples. It is unclear whether bacteria acquired phage resistance or phage hosts were not present at the time of sample collection. Yet, this is another example showing that the majority of phage sequences are to be explored in the coming years [81]. To understand intracommunity interactions in human body inhabited by multiple microorganisms, it is imperative to isolate and characterize phages as they constitute a large and significant subpopulation, including within urobiome. Prior studies have suggested that such interactions do not necessarily have to be beneficial for humans. For example, some *Lactobacillus* phages could play a role in the shift in the vaginal community structure and contribute to bacterial vaginosis [24].

Proper characterization of urinary microbiota requires approaches based on methodology. It has been pointed out by numerous authors that the type of specimen is crucial in selecting the appropriate collection method (voided urine specimens versus catheterization). In addition, the bladder has orders of magnitude less microbial biomass when compared to vagina or gastrointestinal tract, which makes it even more challenging during drawing process [41]. Although there is a dogma saying that phages tend to occur in the vicinity of their hosts, sources describing phage isolation from urine samples remain scarce. Certainly, a metagenomic culture-independent approach will provide more information in this matter.

Currently, there is debate on the possible interconnections between vaginal and urinary tract communities within one organism. Because microbial shifts between the gastrointestinal tract and occurrence of UTIs have already been shown, it is more likely that microbiota within the urinary tract is somehow connected. To support this argument, there are reports indicating that patients with recurrent *Clostridium difficile* colitis had significant reduction in the number of UTIs after fecal transplant [46]. Finally, the hidden presence of phages in human samples may greatly affect microbiological and molecular results [82]. This occurrence cannot be underestimated, especially when analyzing urine samples characterized by significantly lower microbiome biomass when compared to samples of human origin from other niches. The dangers resulting from the phage presence in diagnostic clinical samples were also highlighted by Blanco-Picazo [83]. In 59 urine samples, the authors found 184 divergent phage sequences.

Previously published data suggest that phages can appear in the urine following their administration using different routes and can be transferred from the blood into the epithelial tissue [33,84]. One must be aware that several factors can disrupt such journey in the human body. It has been demonstrated that orally applied phages that are similar to each other, in terms of morphology and resistance to external factors, might differ in their translocation into the bloodstream [85]. Phage penetration may be species-specific, but also depend on adequate human immune response. Natural bacteriophages seem to be naturally optimized to circulate in mammalian bodies escaping rapid neutralization [76]. Further, a biological model based on the coevolution of *E. coli* and the lytic RNA phage (Qβ) revealed a coexistence of phage and its bacterial host. During the test, phage and bacteria were both able to change their phenotypes achieving mutual benefits [44]. All of these factors gathered together may play a meaningful role in shaping the urinary microbiota composition.

Undoubtedly, the role of phages within the urinary tract is of ongoing relevance and importance. We believe that the constantly growing knowledge regarding human microbiome and phage prevalence within the urinary tract will expand our understanding on how to utilize this phenomenon for human well-being. With tools enabling modification of microbiome, the treatment of some diseases, including those beyond antibacterial action of phages, could be more efficient and associated with less burden to the patient.

## Figures and Tables

**Table 1:** In vitro and in vivo studies on phage efficacy in the treatment of urinary tract infections (UTIs).

Target Bacteria	Phage Type	Description	Outcome	Type of Study	Reference
*S. aureus* *E. coli* *Streptococcus* spp. *P. aeruginosa* *P. mirabilis*	Pyophage cocktail (Eliava BioPreparations Ltd., Tbilisi, Georgia) *	Bacterial strains isolated from 118 clinical urine samples from patients planned for resection of prostate were tested against Pyo preparation before and after adaptation (selecting h-mutants with a broader and stronger host–phage interaction).	Before adaptation: 24% strains were sensitive, 16% moderately sensitive, 60% resistant After adaptation: 41% strains were sensitive, 34% moderately sensitive, 25% resistant	in vitro	[47]
*S. aureus* *E. coli* *Streptococcus* spp. *P. aeruginosa* *P. mirabilis*	Pyophage cocktail (Eliava BioPreparations Ltd., Tbilisi, Georgia) *	Nine patients aged 56–85 subjected to resection of prostate received Pyo preparation intravesically two times daily for 7 days, starting the first day after surgery. The solution of 20 mL was retained in the bladder for approximately 30–60 min.	Bacterial count decreased from two to seven orders (below limit of detection) in 6 patients, remained at the same level in 1 patient and in 1 patient *Enterococcus* strain was replaced by *E. coli* post-treatment growth with increased bacterial count.	in vivo	[47]
*Enterobacter cloacae*	Three phages (E-2, E-3 and E-4) isolated from wastewater treatment plant	*E. cloacae* was challenged with the three phages, separately and in a cocktail both in PBS buffer and urine sample at MOI 100.	Three tested phages were effective to inactivate *E. cloacae* in a buffer solution when used individually or, with significantly higher inactivation, in phage cocktails. Inactivation in urine was significantly lower but occurred with phage stability in urine lasting up to 12 h.	in vitro	[60]
UPEC	Three phages (vB_EcoP_ACG-C91, vB_EcoM_ACG-C40 and vB_EcoS_ACG-M12) isolated from sewage which were able to lyse 80.5% of a subset (42) of the UPEC strains able to form biofilms.	UTI *E. coli* isolate Can 91 was selected to determine the phage effectiveness to degrade biofilms because of the isolate’s ability to form strong biofilms in microtiter wells after 48 h and its sensitivity to all the three phages isolated.	All phages significantly reduced the biofilm within 2–12 h of incubation. Correlation was observed between phage sensitivity and specific serotypes of the UPEC strains.	in vitro	[61]
Multidrug-resistant *Klebsiella pneumoniae* isolated from urine	Cocktail of five *Klebsiella* phages (Kp165, Kp166, Kp167, Kp158, Kp169) with lytic coverage to isolated *Klebsiella* strain	A 63-year-old female patient with recurrent UTI with a long history of type 2 diabetes mellitus and hypertension was unsuccessfully treated with phages (phage resistant mutants developed within days)	Phage therapy combined with non-active antibiotics completely eliminated pathogenic strain and the recurrent UTI symptoms subsequently disappeared. No signs of recurrence were observed for this patient under antibiotic-free conditions during 6 months of follow-up.	in vivo	[62]
*Proteus mirabilis* isolated from UTIs	Three lytic *Proteus* phages (ΦRS1-PmA, ΦRS1-PmB, and ΦRS3-PmA) isolated from wastewater treatment plant	*P. mirabilis* cell suspensions were inoculated directly into the in vitro bladder models at either 10^10^ CFU or 10^3^ CFU, representing late-stage or early stage-infection, respectively.	In model with an established infection, a single dose of the phage cocktail (10^10^ PFU; MOI 1) significantly extended the time needed to develop biofilm. In model with early infection: the phage cocktail completely prevented catheter blockage and eradicated infection.	in vitro	[63]
12 *P. aeruginosa* and 10 *P. mirabilis* isolates (almost exclusively clinical and urinary tract related strains)	The anti-*Pseudomonas* cocktail contained six *P. aeruginosa* phages (ΦPaer4, ΦPaer14, M4, 109, ΦE2005-A, and ΦE2005-C,) The anti-*Proteus* phage cocktail contained four *P. mirabilis* phages (ΦPmir1, ΦPmir32, ΦPmir34, and ΦPmir37)	The biofilm formation was determined in a flowing catheter reactor model over a three to four-day period with the use of artificial urine medium (AUM). Phage cocktail against *Pseudomonas* (1 × 10^9^ PFU/mL each) and against *Proteus* (3 × 10^8^ PFU/mL each) were used. Hydrogel-coated catheters were pretreated with one or both cocktails. The bacterial strains used for challenge were grown overnight in AUM and sub-cultured at initial concentration of approx. 1 × 10^3^ CFU/Ml.	Phage pretreatment reduced *P. aeruginosa* biofilm counts by 4 log_10_ CFU/cm2 (*p* ≤ 0.01) and *P. mirabilis* biofilm counts by >2 log_10_ CFU/cm2 (*p* ≤ 0.01) over 48 h.	in vitro	[64]
ESBL-positive *K. pneumoniae*	*Klebsiella* phage obtained from Eliava Institute in Tbilisi, Georgia	58-year-old renal transplant patient with UTI. Phage regimen included oral application and bladder irrigation for four weeks, followed by oral and intravesical application every second day over an eight-week period.	Recurrent UTI by an ESBL-positive *K. pneumoniae* strain was successfully treated with a combination of meropenem and phages after failure of antibiotic therapy applied alone.	in vivo	[65]
*Enterococcus* spp., *E. coli*, *P. mirabilis*, *P. aeruginosa*, *Staphylococcus* spp., and *Streptococcus* spp.	Pyophage cocktail (Eliava BioPreparations Ltd., Tbilisi, Georgia) *	Male patients >18 years of age qualified for prostate resection with complicated or recurrent uncomplicated UTI received intravesically 20 mL of Pyophage or placebo twice daily for 7 days	Phage treatment turned out to be non-inferior to typical antibiotic treatment but was not superior to bladder irrigation (placebo).	in vivo	[66]

* active against a broad spectrum of uropathogenic bacteria: *Staphylococcus aureus*, *E. coli*, *Streptococcus* spp., *Enterococcus* spp., CFU—colony forming unit; ESBL—extended-spectrum β-lactamase; MOI—multiplicity of infection; PFU—plaque-forming unit; UPEC—uropathogenic *E. coli*.
